# Microarray Technology Applied to Human Allergic Disease

**DOI:** 10.3390/microarrays6010003

**Published:** 2017-01-28

**Authors:** Robert G. Hamilton

**Affiliations:** Division of Allergy and Clinical Immunology, Departments of Medicine and Pathology, Johns Hopkins University School of Medicine, Baltimore, MD 21224, USA; rhamilt2@jhmi.edu; Tel.: +1-410-550-2031

**Keywords:** IgE, human, immunoenzymetric assay, immunosorbent allergen chip, ISAC, serodiagnosis, microarray, molecular allergen, allergen extract, component resolved diagnosis

## Abstract

IgE antibodies serve as the gatekeeper for the release of mediators from sensitized (IgE positive) mast cells and basophils following a relevant allergen exposure which can lead to an immediate-type hypersensitivity (allergic) reaction. Purified recombinant and native allergens were combined in the 1990s with state of the art chip technology to establish the first microarray-based IgE antibody assay. Triplicate spots to over 100 allergenic molecules are immobilized on an amine-activated glass slide to form a single panel multi-allergosorbent assay. Human antibodies, typically of the IgE and IgG isotypes, specific for one or many allergens bind to their respective allergen(s) on the chip. Following removal of unbound serum proteins, bound IgE antibody is detected with a fluorophore-labeled anti-human IgE reagent. The fluorescent profile from the completed slide provides a sensitization profile of an allergic patient which can identify IgE antibodies that bind to structurally similar (cross-reactive) allergen families versus molecules that are unique to a single allergen specificity. Despite its ability to rapidly analyze many IgE antibody specificities in a single simple assay format, the chip-based microarray remains less analytically sensitive and quantitative than its singleplex assay counterpart (ImmunoCAP, Immulite). Microgram per mL quantities of allergen-specific IgG antibody can also complete with nanogram per mL quantities of specific IgE for limited allergen binding sites on the chip. Microarray assays, while not used in clinical immunology laboratories for routine patient IgE antibody testing, will remain an excellent research tool for defining sensitization profiles of populations in epidemiological studies.

## 1. IgE and Allergic Disease

IgE antibody was identified in 1967 as the molecular gatekeeper which controls the elicitation of allergic symptoms in humans [1,2]. Antibodies of the IgE isotype are produced by B-cell lymphocytes as a result of the exposure of a genetically-predisposed individual to any of hundreds of allergenic sources. Once produced, IgE antibodies circulate in the blood and bind onto high affinity epsilon specific receptors on mast cells in the skin and basophils in the blood. At this point, an individual can be considered sensitized (IgE antibody-positive) to the particular allergen specificity, although they may not manifest any allergic symptoms [3]. Repetitive allergen exposure induces a heightened immune response with an increase in IgE antibody levels in the blood. At the point where a critical mass of IgE antibody binds to the surface of an individual’s mast cells and basophils, allergen that is inhaled, ingested or injected into the body produces cross-links of surface bound antibodies sufficient to cause mast cells and basophils to become activated and release stored histamine and produce new vasoactive leukotriene mediators. The location of the release of histamine and leukotrienes in the body determines the location (skin, lung, gastrointestinal tract, systemic) and magnitude (severity) of the allergic symptom(s). Localized release in the skin can cause itching, swelling and redness. In contrast, systemic release of mediators can cause anaphylaxis, in some cases leading to death [4].

## 2. Detection of IgE Antibody in Serum

The detection and quantitation of the levels of allergen-specific IgE antibody in human serum was made possible in 1967 with the discovery of IgE as a unique immunoglobulin isotype [1,2]. Purified IgE from a rare IgE myeloma containing serum was used to produce a polyclonal anti-human IgE reagent that was radioiodinated and used as a detection protein for IgE to establish first a singleplex radioisotopic IgE antibody assay called the radioallergosorbent test or RAST [5]. Cellulose paper disks were individually coupled with allergenic proteins from over 100 different allergenic sources (pollens from weeds, grasses and trees; airborne mold spores; animal epidermal proteins, ingested foods; injected venoms and drugs; inhaled insect proteins; and occupational allergens). The addition of serum containing specific antibodies resulted in the binding of all isotypes (IgG, IgA, IgM, IgE) of allergen-specific antibody (if present) from the serum onto the cellulose–antigen solid phase. Following a buffer wash to remove unbound serum proteins, bound IgE was detected with a radiolabeled anti-human IgE conjugate.

## 3. Technological Enhancements Leading to Microarrays

Over the years, significant technological developments have allowed the use of (a) non-isotopic poly- and monoclonal anti-human IgE Fc conjugates to detect bound IgE antibody; (b) the World Health Organization IgE reference preparation [6] to allow calibration of the allergen-specific assay which has enhanced inter-laboratory standardization; (c) new solid phase matrix materials with higher binding capacities for allergenic molecules; (d) engineering advances in robotics and electronics that resulted in current, computer-driven singleplex autoanalyzers; and most recently (e) the production of purified recombinant and native allergenic components [7,8]. These technological developments have resulted in our current state of the art singleplex standalone assays, autoanalyzers that are used throughout the world to measure total and allergen-specific IgE antibodies [7]. A positive IgE antibody response signifies that an individual has become sensitized, which is necessary, but is not sufficient to make the definitive diagnosis of allergic disease [9]. The IgE antibody’s concentration, strength of binding (affinity/avidity), specificity and the percentage of specific IgE to total IgE each plays an interrelated role in translating a humoral IgE antibody response into a clinical allergic symptom [7].

Microarray technology has been only recently applied to the field of diagnostic allergy serology. The first report of chip-based microarray technology being applied to the diagnosis of human allergic disease occurred in 2002. Hiller et al. immobilized 94 purified recombinant and natural allergen molecules at 0.3 mg/mL in 150 mM sodium phosphate buffer in triplicate onto a pre-activated amine reactive coated glass slide [10]. The slide was then used as a solid phase antigen to bind allergen-specific antibodies (principally IgG and IgE) from 200 microliters of human serum diluted 1:5 in an initial incubation. Following a buffer wash to remove unbound proteins, bound IgE was detected with a fluorophore-labeled anti-human IgE in a second incubation step. After a second buffer wash, bound fluorophore was detected in a fluorescent microarray reader and an individual’s IgE antibody specificity profile was obtained. This chemistry of the assay called the immunosorbent allergen chip or ISAC was patterned after the first singleplex radioallergosorbent test (RAST) for allergen-specific IgE antibody reported by Wide et al. [5]. The ISAC is currently the predominant IgE antibody microarray that is available to clinical immunology laboratories.

There are reports of the use of microarray technology in the detection of IgE antibody for a number of special patient care related services. Reported applications include the assessment of poly-sensitization (e.g. detection of IgE antibody to different allergen groups), clarification of the allergen triggers for patients with atopic dermatitis and anaphylaxis and the selection of appropriate allergens for use in immunotherapy [11,12,13,14,15]. However, it is rare to use microarray-based IgE antibody assay (e.g., ISAC) in routine patient testing for several reasons. First, microarrays cannot technically cover all clinically relevant allergen specificities so singleplex assays are additionally required when the patient’s history suggests an offending allergen not in the microarray. Second, the use of a fixed panel of allergens forces testing of IgE antibody to allergen specificities that may not be relevant to the patient based on their clinical history. Because IgE antibody measurements indicate sensitization and not allergic disease [3], this can create a problem that requires interpretation of low level IgE antibody to some allergen specificities that are clinically tolerated by the patient. Third, the panel-based microarray testing is not generally reimbursed by medical insurance and thus is often too expensive for the patient out of pocket for over 100 measurements. Finally, there are technical limitations associated with the microarray-based assays involving analytical sensitivity and specificity, IgG anti-allergen interference and quality control that are noted below which make the singleplex IgE antibody assays those of choice for routine diagnostic allergy testing. 

## 4. Advantages and Limitations of IgE Antibody Microarray Assays.

**Figure 1:**
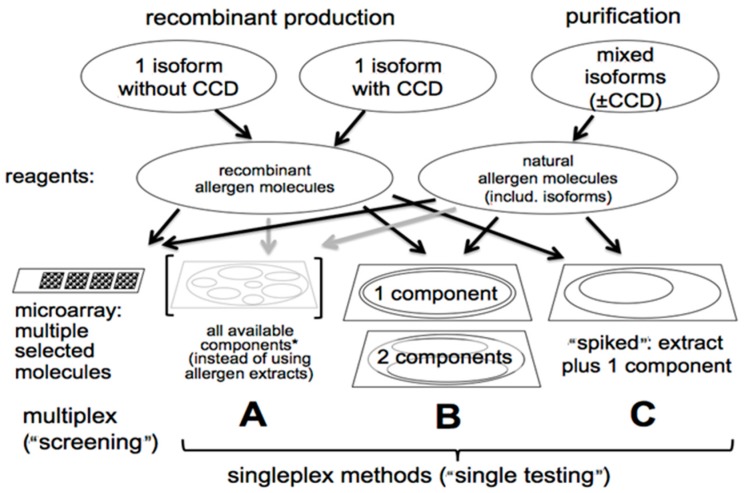
Four assay formats which employ recombinant (with and without carbohydrate cross-reactive determinants or CCDs) and native purified allergens. CCDs confound specificity assessment especially with *Hymenoptera* venom specific IgE assays. Thus, the use of allergenic molecules without CCDs aid in more accurately defining the true allergenic specificity of the IgE antibody response. The microarray multiplex format (left assay) is used by the immunosorbent allergen chip or ISAC. It involves 112 individual allergens in triplicate arrays that are immobilized on an amine-activated chip. * A mixture of components from a single allergen specificity can be used to mimic an unpurified extract that often has extraneous non-allergenic material together with allergens. The arrows indicate the type of allergosorbents where recombinant and natural allergenic molecules can be effectively used. Adapted with permission from [8].

Figure 1 depicts a number of possible singleplex and microarray assay configurations that can be employed with recombinant and natural allergen molecules. The most attractive microarray configuration involves spotting purified allergens individually on activated glass chips for multiple specificity IgE antibody screening. Most recently, the current ISAC assay has 112 individual allergenic molecules spotted in triplicate. A research version of this assay called the Mechanisms of the Development of ALLergy (MeDALL) Allergen Chip expanded the number of allergenic molecules immobilized on the chip to 170 [16]. It was used to study IgE antibody sensitization profiles of European birth cohorts. Certainly, the principal advantage of the multiplex microarray chip is its impressive technology that permits the detection of IgE antibody to a broad spectrum of clinically relevant allergenic molecules using a small quantity of serum. As suggested above, there are limitations to the microarray-based panel testing format. It encourages abuse by performing measurements of unwanted or unneeded IgE antibody specificities that are not indicated by the patient’s clinical history. Moreover, the chip-based microarray is less quantitative and potentially less analytically sensitive than the singleplex autoanalyzers that are in widespread use throughout the world. Chip-based microarrays are more difficult to quality control due to the number of different allergens on a single solid phase surface. They pose an increased risk for greater inter-lot variability due to the need to perform simultaneous quality control on over 100 different allergen molecules (or extracts). Possibly of most concern, chip microarrays are subject to interference by allergen-specific IgG antibodies due to the low level of allergen deposited on the chip per spot. This can compromise the quantitative accuracy and bias the analysis toward only high affinity IgE antibody. Alternatively, the interference can be viewed positively as a surrogate marker for the result of specific immunotherapy that elicits IgG-blocking antibody [17].

The advantage of the IgE antibody chip-based microarray rests in its conservation of serum volume, increased speed of analysis, reduced technician intervention and the use as an optimal configuration for point-of-care tests. The availability of recombinant allergenic molecules has enhanced IgE antibody assay performance in a number of ways. First, it has permitted an increased analytical sensitivity by supplementing extracts that are deficient in a particular allergen specificity (e.g., Cor a 1 for hazelnut; Hev b 5 for *Hevea brasiliensis* natural rubber latex). Second, there is an increase in analytical specificity when selected molecules are used to detect IgE antibodies. For instance, Ara h 2 for peanut; Cor a 9 and 14 for hazelnut detect IgE antibody profiles that aid in determining a clinical risk for a systemic reaction and clarifying the need for an oral food challenge. Third, molecular allergens allow the identification of cross-reactivity (e.g., pan allergens Phl p 7 [polcalcin] and Phl p 12 (profilin) in Timothy grass, lipid transfer proteins in plant food and pollens [18]). And fourth, selected molecules define IgE antibodies as a marker of a genuine primary sensitization. (e.g., Fel d 1 for cat; Ves v 1 and Ves v 5 for yellow jacket venom). The microarray format allows simultaneous IgE antibody testing of multiple structurally similar allergens and thus allows more time and cost-effective evaluation of possible IgE antibody binding to the principal families of cross-reactive allergen molecules (e.g., Pathogenesis-Related proteins (PR10 family), tropomyosins, serum albumins, non-specific lipid transfer proteins, profilins, polcalcins, and seed storage proteins).

## 5. Alternative Microarray Assays for IgE Antibody

While the ISAC was the first and it has been the most visible microassay for the detection of IgE antibody, other groups have reported different assay formats. Unlike the ISAC that is a manual assay, Williams et al. reported an automated microarray system called the Microtest that involves the use of a self-contained biochip and reagent cartridge [19]. It measures IgE antibody specific for 19 common airborne and food allergen extracts and 16 allergenic components. Its limitation is a fixed allergen menu and the need for 100 microliters of serum that is applied over a surface area containing 100 spots per cm^2^. Agreement studies with the ISAC, singleplex ImmunoCAP and skin prick testing involving the most prevalent allergen specificities from cat, dog, mite, Timothy grass, Birch tree and peanut produced correlation coefficients between 0.73 and 0.95.

The multiplex Luminex xMAP technology-based microarray reported by King involves a magnetic xMAP bead set with a discrete number (*n* = 7 to 16) of immobilized purified indoor areoallergens [20]. These are used to bind antibodies from human serum and once bound, IgE antibody is subsequently detected with a biotin anti-IgE and Strepavidin-conjugated phycoerythrin. Correlation with an Enzyme-Linked ImmunoSorbent Assay (ELISA) and fluorescent enzyme immunoassay ranged from R = 0.44 to 0.94. It’s advantage was the use of 20 microliters of serum per analysis and its good general reproducibility. The limited allergen specificity repertoire restricts this assay to principal research use.

Renault et al. report a microarray that uses multiple food extracts immobilized on their FAST slides with 4800 dots per slide in triplicate [21]. They prepared their extracts by homogenizing foods, defatting them with hexane and extracting them in phosphate buffered saline containing triton, dithiothreitol, 2% glycerol. They normalized based on total protein and imprinted 350 food extracts onto their activated slides which were then dried, blocked with 1% bovine serum albumin and then used to detect IgE, IgA, IgM and IgG antibodies in sera from allergic patients. General concern with this assay is the immobilization of heterogeneous, uncharacterized extracts on small chip surface areas that raises questions about what actual allergen specificities are immobilized, the working range of the assay, the extent of interference caused by the presence of non-IgE antibody, general concerns about the accuracy of their measurements and the difficulty with inter-lot quality control for variability.

## 6. Concluding Thoughts

The combination of availability of molecular recombinant and native allergens and the microchip technology has fostered in the use of microarrays that can simultaneously measure IgE antibodies to as many of 112 individual allergen specificities with small quantities of serum (e.g., 40 microliters). The use of molecular allergens can enhance the limit of quantitation of an IgE antibody assay by supplementing allergens that are missing or in low abundance in extracts. They can enhance analytical specificity by detecting IgE antibodies to particular stable allergens that signal a high risk for a systemic allergic reaction. They can identify IgE antibody responses to cross-reactive allergens present in foods and pollens. Finally molecular allergens can identify genuine primary sensitization which is not possible with the use of allergen extracts. IgE antibody microarrays have the advantage of increased speed of analysis with reduced turn-around time and conservation of sample volume which is attractive for pediatric testing. The microarray assays tend to be simpler in design, with fewer reagents that can reduce cost and technician time. All these advantages come with the constraint of a lower analytical sensitivity, more complex quality control, and less quantitative results than are generated by singleplex IgE antibody assays. The panel format of the chip forces IgE antibody analyses to all 112 allergens on the microarray which can encourage abusive testing that results in the measurement of unneeded or unwanted IgE specificities. Finally, due to the limited amount of allergenic protein that is deposited on the chip per dot, high levels of allergen-specific IgG antibody may interfere by competing with the binding of IgE antibody of the same specificity, and thus reduce the accuracy of IgE antibody measurements. The overall conclusion is that the current diagnostic microarray assays for IgE antibody will remain excellent research tools for epidemiological studies. They will, however, not compete in the clinical laboratory arena with singleplex assays that have superior analytical sensitivity, quantitation and the ability to use allergen extracts that are viewed as comprehensive in terms of containing the principal allergens in any given allergen specificity.
